# Risk of thyroid neoplasms in patients with 22q11.2 deletion and DiGeorge-like syndromes: an insight for follow-up

**DOI:** 10.3389/fendo.2023.1209577

**Published:** 2023-08-10

**Authors:** Walter Maria Sarli, Silvia Ricci, Lorenzo Lodi, Federica Cavone, Lucia Pacillo, Carmela Giancotta, Graziamaria Ubertini, Giampiero Baroncelli, Caterina Cancrini, Chiara Azzari, Stefano Stagi

**Affiliations:** ^1^ Department of Health Sciences, University of Florence, Florence, Italy; ^2^ Paediatric Immunology Division, Meyer Children’s Hospital IRCCS, Florence, Italy; ^3^ Pediatrics Unit, Department of Clinical and Experimental Medicine, University of Pisa, Pisa, Italy; ^4^ Unit of Clinical Immunology and Vaccinology, Bambino Gesù Children’s Hospital IRCCS, Rome, Italy; ^5^ Research Unit of Primary Immunodeficiency, Bambino Gesù Children’s Hospital IRCCS, Rome, Italy; ^6^ Department of Systems Medicine, University of Rome “Tor Vergata”, Rome, Italy; ^7^ Unit of Endocrinology and Diabetology, Bambino Gesù Children’s Hospital IRCCS, Rome, Italy; ^8^ Auxoendocrinology Division, Meyer Children’s Hospital IRCCS, Florence, Italy

**Keywords:** thyroid cancer, thyroid nodules, 22q11 deletion syndrome, DiGeorge, genetic syndrome

## Abstract

**Introduction:**

The chromosome 22q11.2 deletion syndrome comprises phenotypically similar diseases characterized by abnormal development of the third and fourth branchial arches, resulting in variable combinations of congenital heart defects, dysmorphisms, hypocalcemia, palatal dysfunction, developmental or neuropsychiatric disorders, and impairment of the immune system due to thymic dysfunction. Other genetic syndromes, often called DiGeorge-like, share clinical and immunological features with 22q11.2 deletion syndrome. This syndrome has been rarely associated with malignancies, mainly hematological but also hepatic, renal, and cerebral. Rarely, malignancies in the head and neck region have been described, although no aggregate of data on the development of thyroid neoplasms in patients with this clinical phenotype has been conducted so far.

**Materials and methods:**

To characterize this possible association, a multicenter survey was made. Thus, we present a case series of five pediatric patients with 22q11.2 deletion syndrome or DiGeorge-like syndrome who were occasionally found with confirmed or highly suspected neoplasms of the thyroid gland during their follow-up. In three cases, malignancies were histologically confirmed, but their outcome was good due to an early recognition of suspicious nodules and precocious surgery.

**Conclusions:**

This study underlines for clinicians the higher risk of neoplasms in the head and neck district for patients affected by these syndromes. It also emphasizes the importance of a prolonged clinical and ultrasound follow-up for patients with this clinical and immunological phenotype.

## Introduction

The chromosome 22q11.2 deletion syndrome (22q11.2DS) is caused by a hemizygous deletion that is *de novo* in more than 90% of cases (1). In addition, 22q11.2DS encompasses several phenotypically similar diseases that share abnormal development of the third and fourth branchial arches, resulting in variable combinations of congenital heart defects, dysmorphisms, hypocalcemia, palatal dysfunction, developmental delay, neuropsychiatric disorders, and impaired immune function due to thymic hypoplasia or aplasia (2).

The most famous of these diseases is DiGeorge syndrome (DGS) that is usually caused by the loss of the *TBX1* gene (MIM_188400), located in the 22q11 region, which is an important transcription factor necessary for the development of the thyroid, parathyroids, palate, teeth, thymus, and heart (2). Sometimes, DGS is not caused by 22q11.2 deletion but *TBX1* pathogenic mutations (3). Nevertheless, there are many other genetic syndromes, often called DiGeorge-like, with clinical features like DGS but other chromosomal deletions, such as 10p13 (MIM_266500), 17p13.1 (MIM_613776), 16p11.2 (MIM_611913), and 4q34.1q35.2 (4–7), or others within the context of CHARGE (MIM_214800) or Opitz G/BBB syndrome (MIM_300000) (8, 9). However, in 6%–17% of patients, a genetic cause remains unknown (10).

Thyroid anomalies are frequently discovered in 22q11.2DS. Approximately half of the patients have structural anomalies such as thyroid hypoplasia, absent isthmus, and abnormal extension probably due to *TBX1* haploinsufficiency (11–13). Thyroid dysfunction can also be caused by autoimmunity. It is estimated that over 8% of patients with 22q11.2DS will develop autoimmunity with age (14). Recently, we described 73 children with 22q11.2DS, followed up for 9.51 ± 5.72 years. Totally, 21.9% developed autoimmune thyroid disease (ATD) before the age of 18, including 20.5% Hashimoto’s thyroiditis (HT) and 1.4% Graves’ disease (GD) (15). Among 22q11.2DS patients who developed ATD, one case of thyroid cancer in one adolescent developing GD was described, strongly recommending periodic screening with ultrasound scan in these patients (15).

It is known that many congenitally inherited syndromes are associated with a higher risk of malignancy (16, 17) with the development of malignancies, mainly hematological but also hepatic, renal, and cerebral (8, 18). Malignant tumors in the head and neck region, as well as in other districts, have rarely been described (19–21). The risk of malignancy in 22q11.2DS is moderately increased when compared to the healthy general pediatric population with a reported frequency of 1% in the 22q11.2DS population (20). A recent Finnish nationwide register-based cohort study by Wahrmann et al. (22) described a 2.1% rate of malignancy in 98 patients with 22q11.2DS.

Very few cases of patients with 22q11.2DS and thyroid neoplasms, benign or malign, have been described so far (8, 14, 19). On the other hand, to the best of our knowledge, no cases of thyroid neoplasms have been reported in patients with DiGeorge-like syndromes.

To characterize this possible association, a multicenter survey was made. Thus, we present a case series of five pediatric patients with 22q11.2DS or DGS-like who were occasionally found with confirmed or highly suspected neoplasms of the thyroid gland during their follow-up.

## Materials and methods

We performed a retrospective multicenter study of patients with 22q11.2DS or DGS-like who presented with thyroid or parathyroid neoplasms before the age of 18 between 4 January 1985 and 6 July 2022. The study recruited patients with a confirmed genetic diagnosis of 22q11.2DS or with a DGS-like, based on a highly evocative clinical and laboratory phenotype, from the Paediatric Immunology Division and Auxo-endocrinology Division of Meyer Children’s Hospital in Florence (University of Florence), Santa Chiara’s Hospital in Pisa (University of Pisa), and Bambino Gesù Children Hospital in Rome (University of Roma Tor Vergata), Italy. The Tobias and the European Society of Immunodeficiencies (ESID) criteria were initially used to evaluate patients’ susceptibility to genetic analysis for 22q11.2DS (23, 24). All patients were monitored annually or biannually up to the age of 18 through clinical assessments and thyroid ultrasounds and blood tests to measure thyroid function and autoantibodies. A total of three patients with 22q11.2DS and confirmed thyroid cancers were extensively described below.

The study was conducted according to the Declaration of Helsinki II. Written informed consent from the patients’ parents or legal guardians was acquired before data collection. Patient data were retrospectively retrieved from the clinical records and anonymously collected in an Excel^®^ spreadsheet. A specific approval by the local ethical committee was not required because all analyses included in this study were performed as part of routine clinical activity according to Good Clinical Practice.

Clinical, ultrasound, and histological data were collected from the clinical records of all patients during the follow-up. Ultrasound scans were performed with a linear multifrequency transducer. An extended immunologic phenotype was performed. All values were evaluated by standard methods and compared with age-matched normal values.

## Results

**Figure 1:**
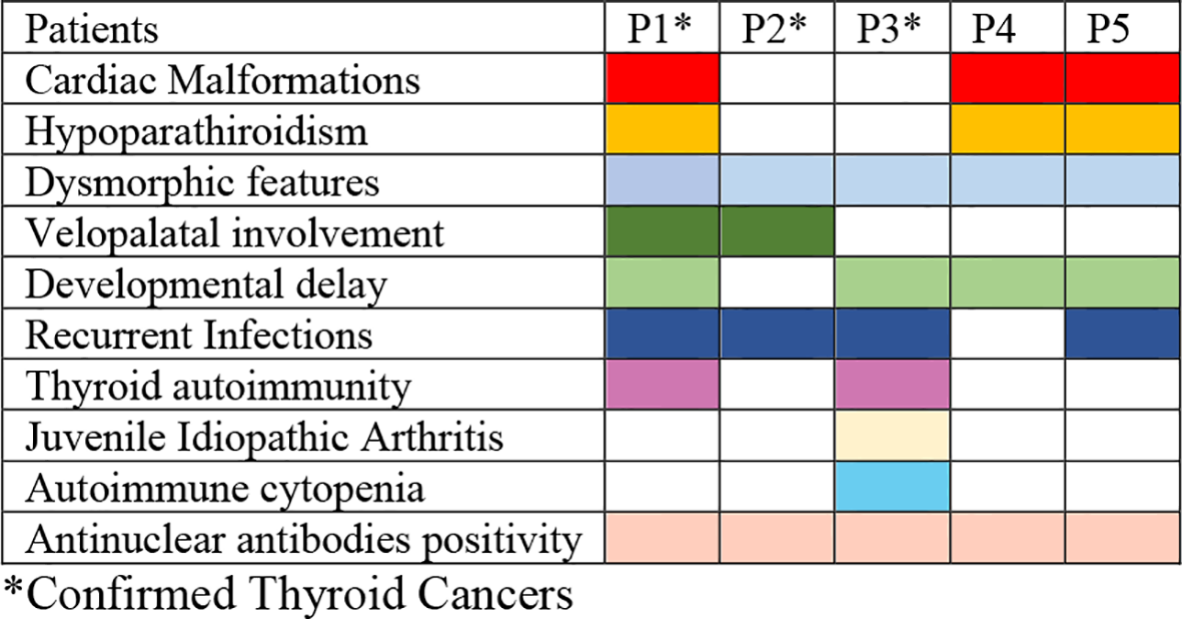
Graphical representation of the main clinical features of the patients. *Confirmed thyroid cancers.

**Figure 2:**
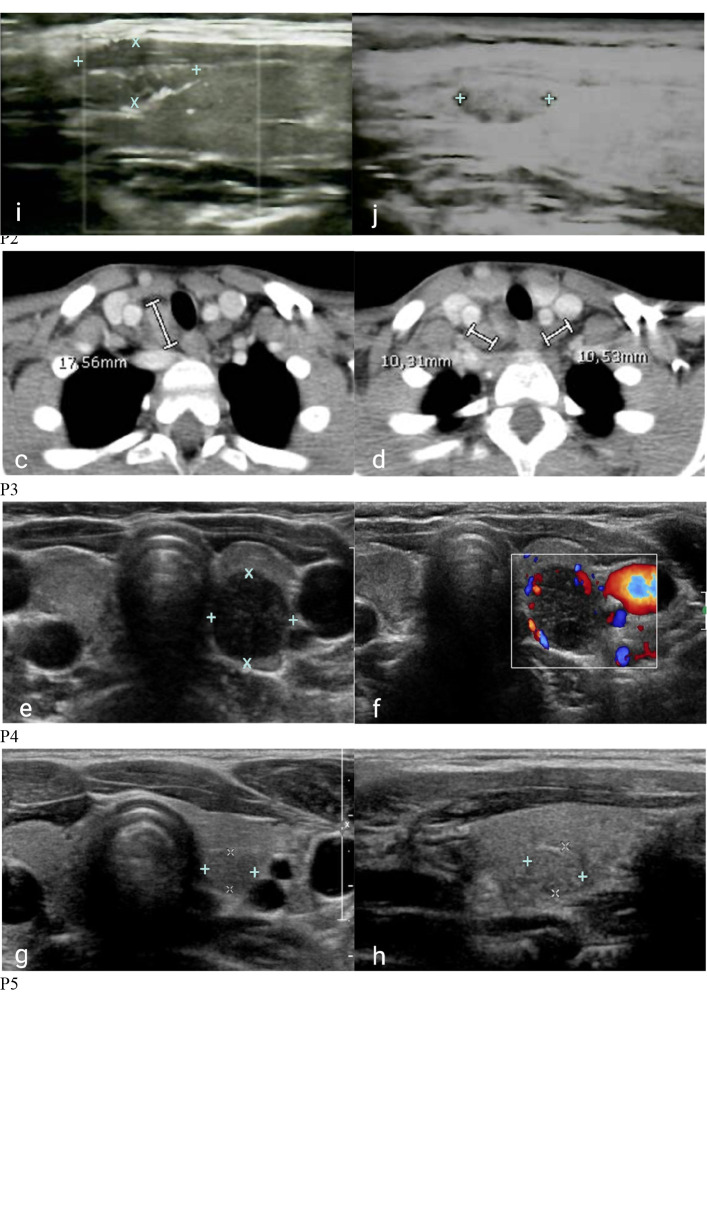
Cervical ultrasonography and computerized tomography of patients with 22q11DS and DiGeorge-like syndrome. P1. Inhomogeneous hypoechoic nodule in the right lower lobe in coronal **(A)** and sagittal **(B)** ultrasound projections. P2. Metastatic involvement of right lateral cervical lymph nodes **(C)** and normal lymph nodes **(D)** in coronal CT. P3. Inhomogeneous nodule with hypoechogenic areas **(E)** and peripheral vascular overflow **(F)** in the left lobe in coronal ultrasound projections. P4. Roundish hypoechoic nodule in the posterior region of the left lobe in close proximity to the carotid artery in coronal **(G)** and sagittal **(H)** ultrasound projections. P5. Iso-hypoechoic nodule in the upper third of the right lobe with blurred margins, and intra- and perilesional vascularity in sagittal ultrasound projections in sagittal ultrasound projection: first identification **(I)** and 1-year follow-up **(J)**.

Between 4 January 1985 and 6 July 2022, a total of 275 patients with 22q11.2DS (260 patients) and DGS-like (15 patients) were consecutively observed in the three centers for multidisciplinal follow-up and periodic thyroid ultrasound scans. During follow-up, thyroid malignancies were found by ultrasound scans in three patients with 22q11.2DS (two boys and one girl) with a median age of 10.30 years ± 1.48 years at cancer diagnosis. None of them was previously exposed to radiation (**Figure 1**). None of them had a family history of thyroid or parathyroid carcinoma or adenoma. Two patients had autoimmune hypothyroidism and hyperthyroidism, respectively, at the time the nodules were discovered. The mean maximum diameter of confirmed malignant nodules was 23.16 ± 15.18 mm. According to the classification of the American Thyroid Association (ATA), all three nodules showed ultrasound characteristics of a high risk of malignancy (25). The clinical, ultrasound, and immunophenotypic characteristics of the patients are described in **Figure 2** and **Tables 1**, **2**.

**Table 1:** Lymphocyte subsets of the patients.

Patient	P1*	P2*	P3*	P4	P5
Lymphocyte (10^3^/µL)	2.268 2.500 (1.662 – 3.448)	**1.324** 2.285 (1.340 – 3.173)	**1.040** 2.500 (1.662 – 3.448)	2.773 2.500 (1.662 – 3.448)	**1.411** 3.800 (2.340 – 5.028)
CD3 (10^3^/µL)	**1.176** 1.793 (1.239 – 2.611)	**0.281** 1.629 (0.954 – 2.332)	**0.623** 1.793 (1.239 – 2.611)	1.011 1.629 (0.954 – 2.332)	**0.558** 1.793 (1.239 – 2.611)
CD3CD4 (10^3^/µL)	**0.335** 1.030 (0.646 – 1.515)	**0.149** 0.887 (0.610 – 1.446)	**0.351** 1.030 (0.646 – 1.515)	0.705 0.887 (0.610 – 1.446)	**0.339** 1.030 (0.646 – 1.515)
CD3CD8 (10^3^/µL)	0.465 0.595 (0.365 – 0.945)	**0.114** 0.518 (0.282 – 0.749)	**0.205** 0.595 (0.365 – 0.945)	**0.262** 0.518 (0.282 – 0.749)	**0.187** 0.595 (0.365 – 0.945)
CD19 (10^3^/µL)	0.593 0.403 (0.276 – 0.640)	0.367 0.321 (0.173 – 0.685)	**0.215** 0.403 (0.276 – 0.640)	0.468 0.403 (0.276 – 0.640)	**0.256** 0.403 (0.276 – 0.640)
CD3CD16CD56 (10^3^/µL)	0.335 0.262 (0.120 – 0.483)	0.296 0.230 (0.087 – 0.504)	0.236 0.262 (0.120 – 0.483)	0.311 0.230 (0.087 – 0.504)	**0.126** 0.403 (0.276 – 0.640)
CD4CD45CD31 (%)	**24%** 55% (43% – 67%)	**5%** 49% (37% – 62%)	**12,5%** 49% (37% – 62%)	**35%** 55% (43% – 67%)	50% 55% (43% – 67%)
CD4CD45RA+ (%)	**27%** 65.0% (53.3%–74.0%)	**12%** 54.3% (40.9%–65.7%)	15% 54.3% (40.9%–65.7%)	76% 65.0% (53.3%–74.0%)	45% 65.0% (53.3%–74.0%)
CD4CD45RO+ (%)	**73**% 29.0% (22.1%–36.6%)	**88**% 35.7% (25.1%–52.1%)	**85**% 35.7% (25.1%–52.1%)	24% 29.0% (22.1%–36.6%)	**55%** 29.0% (22.1%–36.6%)
CD19CD27+ (%)	**3.74%** 16.5% (12.7% - 24.5%)	**1.5%** 12.7% (7.9% - 19.8%)	**3.2%** 16.5% (12.7% - 24.5%)	**3.06%** 16.5% (12.7% - 24.5%)	**2.88%** 16.5% (12.7% - 24.5%)
CD27+IgD+ (%)	**2.32%** 10% (7.5% – 12.4%)	**0.25%** 7.3% (4.6% - 10.2%)	**0,76%** 10% (7.5% – 12.4%)	**1.13%** 10% (7.5% – 12.4%)	**1.96%** 10% (7.5% – 12.4%)
CD27+IgD- (%)	**1.42%** 6.5% (5.2% - 12.1%)	**1.25%** 5.4% (3.3% - 9.6%)	**0.99%** 6.5% (5.2% - 12.1%)	**1.93%** 6.5% (5.2% - 12.1%)	**0.92%** 6.5% (5.2% - 12.1%)

The absolute and/or relative numbers of cell subsets are indicated for each patient (upper line). Lower lines indicate normal absolute or relative values for age (26–28).

*Confirmed thyroid cancers. Bold values are those that deviate from normal values.

**Table 2:** Ultrasound features and laboratory details of patients with 22q11.2DS and DiGeorge-like syndrome and thyroid neoplasms.

	P1*	P2*	P3*	P4	P5
Thyroid dimension (mL)	6.79 (2.05 - 9.17)	15.08 (1.20 - 7.09)	5.55 (1.20 - 7.09)	3.41 (2.05-9.17)	10.21 (4.04-13.19)
Nodule Dimension (mm)	14*19*6	40*30*20	12*10.5*9.5	7*5*7.5	9.8*6.7*5
Overflow	–	–	+	+	+
Calcifications	–	+	–	–	–
Inhomogeneity	+	+	+	+	–
Hypoechogenicity	+	+	+	+	–
Lymph Nodes	–	+	+	+	–
TSH (mcU/mL)	0.005 (1.12 – 5)	1.85 (0.8 – 3.5)	150 (1.12 – 5)	2.11 (0.8 – 4)	2.85 (0.8 – 4)
fT4 (ng/mL)	2.38 (1.01 -1.63)	1.53 (1 – 1.9)	0.18 (1.01 -1.63)	1.05 (0.7 – 1.8)	1.09 (0.8 – 1.8)
TG-Ab	–	–	29.9	–	–
TPO-Ab	–	–	2738	41.3	–
TSHR-Ab	9.47	–	–	–	–

The thyroid volume was calculated with Pi/6 × length × width × depth for each lobe and then by adding the two values. Lower line indicates normal values (min–max) for age (29).

Worst laboratory values (lowest or highest) are represented.

*Confirmed thyroid cancers.

### Case 1

P1 is a 12-year-old boy with 22q11.2DS diagnosed through comparative genomic hybridization array (CGH-array) and fluorescence *in situ* hybridization (FISH) at age 9 for a history of speech delay, palatal insufficiency, and submucosal cleft palate. Initial cardiologic evaluation identified pervious foramen ovale (PFO) and aortic root ectasia. During follow-up, his growth was good, and he did not complain of relevant infections. At age 11, routine blood tests for thyroid function and autoimmunity showed decreased thyroid-stimulating hormone (TSH) levels and significantly elevated TSH receptor (TSHR) antibodies. Ultrasound scans revealed diffuse thyroid gland overflow, leading to a diagnosis of Graves’ disease (GD). Treatment with Tapazole was initiated, resulting in good disease control without side effects. However, subsequent ultrasound scans performed after 6 and 9 months revealed diffuse inhomogeneity and a hypoechogenic nodule in the right lower lobe (14 × 19 × 6 mm), indicative of a potential thyroid abnormality. Fine-needle aspirate biopsy (FNAB) was conducted, identifying TIR3b cytology, and total thyroidectomy was performed. Histologic examination confirmed the presence of follicular carcinoma, which was classified as T1N0M0 according to TNM staging. Due to the early diagnosis, the patient did not require adjuvant radiometabolic therapy and is currently disease-free with substitutive therapy.

### Case 2

P2, a 24-year-old man, underwent surgery for intestinal occlusion due to malrotation at 6 days of age. The diagnosis of 22q11.2DS was prompted by dysmorphic features and an interventricular defect and confirmed at 1 month of age through FISH. During the follow-up, multiple thyroid ultrasound scans were performed, all yielding negative results. At the age of 9, a routine ultrasound scan revealed a solid nodular formation completely occupying the right thyroid lobe (40 × 30 × 20 mm). The nodule exhibited hyperechogenic spots in the peripheral zone and inhomogeneous hypoechogenicity in the central zone. Additionally, a right cervical lymphadenopathy was identified, with hyperechogenic spots. FNAB identified a TIR4 phenotype; thus, total thyroidectomy with cervical right lymphadenectomy was performed. Histologic examination revealed papillary thyroid carcinoma infiltrating the peri-thyroid lax tissues, which was classified as T3N1bMx due to metastatic involvement of four right recurrent lymph nodes (NR1, NR3, NR4) and three right lateral cervical lymph nodes (N1, N2). After 5 months, recurrence of disease was observed at the right lateral cervical site (right mandibular angle), necessitating additional surgery and radiometabolic therapy. P2 is currently disease-free and continues to receive care from an adult immunology center.

### Case 3

P3 is an 18-year-old woman with 22q11.2DS diagnosed through FISH at the age of 12 months for dysmorphic features and neurodevelopmental delay. Recurrent respiratory infections were observed since early childhood. At age 3, she was diagnosed with ATD based on increased TSH levels and positive autoantibodies. Substitution therapy with levothyroxine was initiated. Of note, familiar history was positive for autoimmune diseases (aunt with ATD and grandmother with Sjögren’s syndrome). During the follow-up, multiple thyroid ultrasound scans were performed. At the age of 10, a routine ultrasound scan revealed an inhomogeneous nodule (12 × 10.5 × 9.5 mm), with hypoechogenic areas, peripheral vascular overflow, and a suspected lymph node (long axis 10.5 mm). The first FNAB identified a TIR1 cytology, but 5 months later, the second FNAB identified a TIR3B phenotype. Consequently, hemithyroidectomy was performed. Histologic examination identified a papillary thyroid carcinoma Warthin-like variant (pTIb). Histology also showed lymphocyte infiltration as for Hashimoto thyroiditis, while no signs of metastases were found in lymph nodes. She subsequently underwent total thyroidectomy and then radiometabolic therapy with 131-I for ablation of residual thyroid tissue. Later, she remained stably disease-free with substitutive therapy.

Of note, it is worth mentioning that two additional patients with suspected thyroid malignant nodular lesions were identified during their follow-up. The first patient is a boy with 22q11.2DS who is under close surveillance with serial ultrasounds due to the presence of a slightly growing suspicious thyroid nodule that cannot be biopsied due to its proximity to the carotid artery (P4) while awaiting a more radical intervention in case of worsening ultrasound findings. The other patient is a girl with DGS-like who underwent FNAB and right lobectomy for a suspected malignant nodular lesion, which was subsequently identified as Hurtle cell adenoma (P5). Their clinical, ultrasound, and immunophenotypic characteristics are described for comparison and completeness alongside those of patients P1, P2, and P3 in **Figure 2** and **Tables 1**, **2**.

## Discussion

Our results seem to suggest that patients with 22q11.2DS and probably DGS-like can develop a broad range of thyroid neoplasms with a higher degree of malignancy. It is known to date that the prevalence of pediatric thyroid diseases of surgical interest, particularly thyroid cancer, is a rare entity and significantly lower than that of adults (30). Epidemiological surveillance data described 1.2 cases per 100,000 patients under the age of 20 but 0.4 cases per 100,000 in patients aged under 15 (31). However, the incidence of pediatric thyroid nodules and cancer appears to be steadily increasing and associated with a worse prognosis (32), far higher than the expected value in the adult population (5%) (33). In clinical practice, the management of pediatric thyroid nodules largely follows the adult thyroid guidelines (25), with some peculiarities related to pediatric age.

Although limited, our data appear to indicate that 22q11.2DS patients have a significantly higher prevalence of thyroid cancers, confirming the suggestions of the literature (34, 35) and strongly recommending periodic screening also with thyroid ultrasound scan in these patients. In fact, considering only malignant thyroid neoplasms confirmed by histological investigation through a long follow-up and periodic routine thyroid ultrasound scans, a total of three cases of malignant thyroid nodules (P1, P2, and P3) were identified out of a total of 260 patients with 22q11.2DS (1.15%).

Although not specifically intended for drawing conclusion on the thyroid cancer rate, we chose to mention in this case series two patients with 22q11.2DS and DGS-like phenotype who exhibited suspicious thyroid nodules during their follow-up to raise awareness among physicians about the potential risk associated with these conditions.

Indeed, the incidence of thyroid malignancy in patients with 22q11.2DS may be underestimated. This is exemplified by the case of P4, who presents a thyroid nodule with suspicious features of malignancy but cannot be biopsied. Patients with DGS-like phenotype may also have an increased risk of developing thyroid neoplasms, but due to the lack of specific literature on neoplasms in DGS-like patients, this association may have been overlooked. It is important to note that many DGS-like patients remain undiagnosed and thus do not receive comprehensive immunologic and endocrinologic follow-up. However, given their shared clinical and immunophenotypic features with 22q11.2DS patients, some centers adopt a similar follow-up approach for both groups. Our findings highlight the importance of regular ultrasound monitoring in DGS-like patients, as exemplified by the detection of a suspicious nodule in P5, although not confirmed as malignant.

To date, less is known about why patients with clinical phenotypes including 22q11.2DS, and probably DGS-like too, may have an increased risk of malignancies. One of the reasons could lay on the T-cell defects that are common in 22q11.2DS and in DGS-like too (10, 36, 37). As widely described for HIV infection, T lymphopenia or reduced natural killer cells, as well as their dysfunction, may predispose to poor surveillance against neoplastic cells. T-cell dysfunction can also lead to B-cell hyperproliferation and overactivation resulting in a higher risk of developing lymphomas (38, 39). In fact, cases of B lymphoma in patients with 22q11.2DS and severe T-cell impairment have been reported in the literature in the past (40, 41). It is unclear whether the few thyroid cancers described to date in patients with 22q11.2DS were associated with T lymphopenia (34, 42). Generally, there is limited understanding regarding the relationship between the development of thyroid adenomas or carcinomas and lymphopenia. A recent work by Rabold et al. (43) found that T-cell lymphopenia in patients with thyroid cancer indicates an aggressive tumor behavior and might badly influence the outcome of the disease. Lymphopenia could play a role also for persistent infections by oncogenic viruses. As proof for this, there is increasing evidence in the literature on the association between viral infections and thyroid cancer development. Recent work by Moghoofei et al. (44) described the detection of Epstein Barr virus (EBV) DNA in 71.9% of 57 thyroid tumor specimens and thus demonstrated that persistent EBV infection could be associated with an increased risk of thyroid cancer. This evidence was confirmed by a systematic literature review conducted in 2020 by Mostafaei et al. (45) who demonstrated the potential pathogenetic association with several viral infections, not only EBV.

All patients (P1, P2, and P3) with 22q11.2DS who developed thyroid cancer had T lymphopenia, especially in the CD3CD4^+^ and CD3CD8^+^ compartments at immunophenotype analysis. Variable degrees of T lymphopenia were also found in P4 and P5 as described in **Table 1**.

Probably, this may have played a role in cancer development, but we cannot assume that T lymphopenia alone was their only risk factor because there are numerous inborn errors of immunity with profound T-cell deficiency in which an increased risk of thyroid neoplasms is not reported in the literature.

Thus, likely in addition to lymphopenia, there could be other reasons related to the syndrome that we do not fully understand to date but that could contribute to the risk of developing thyroid cancers.

Since allelic losses of chromosome 22q arm were described by molecular analysis of well-differentiated thyroid cancer specimens (46), authors have tried to emphasize the role of this chromosomal region loss in thyroid cancer predisposition. For instance, atypical deletions could involve the tumor suppressor gene *SMARCB1* (MIM_609322), which is associated with rhabdoid tumors, or *DGCR8* gene (MIM_609030), which has been found to result in aberrant levels of miRNA leading to an increased susceptibility to malignancies (47, 48). However, if the reason was related only to this, many more cases of thyroid tumors among patients with 22q11.2DS would have been described so far.

Moreover, this pathogenesis cannot explain why patients with DGS-like, who do not have 22q deletion, may eventually share an increased risk.

Since, in 22q11.2DS, the development of the third and fourth branchial arches from which the thyroid gland derives is impaired (2), an embryological role in predisposition to thyroid cancer cannot be ruled out. Recently indeed, a model of fetal carcinogenesis of thyroid tumors has been proposed (49). According to this model, within a differentiated thyroid gland, there would exist three different types of fetal thyroid cells that could give rise to thyroid cancer cells. Finally, it is necessary to consider thyroid autoimmunity. It is well known that patients with 22q11.2DS have an increased risk of autoimmunity, both thyroid and non-thyroid (2). According to Shugar et al. (50), there is a higher rate of hypothyroidism and hyperthyroidism in children with 22q11.2DS than in the general population. However, as demonstrated in our previous work, through a long ultrasound and endocrinological follow-up, thyroid autoimmunity could be detected in up to 21.9% of patients with 22q11.2DS (15). Moreover, sometimes also ultrasound changes could precede the rise of thyroid autoantibodies or hormone impairment.

A recent study by Montin et al. (51) in patients with 22q11.2DS showed that certain immunologic features, including reduced numbers of recent thymic emigrants (RTEs), reduced naive T cells, and reduced B memory switched, are associated with the development of hematologic autoimmunity. This was also demonstrated in a study by Ricci et al. (52). Much evidence suggests that inflammation induced by thyroid autoimmunity may promote tumor development, although the precise mechanism has not been identified. Nevertheless, the presence of a proinflammatory environment, variable expression of transcriptional regulators, and increased TSH values together could promote tumor development or growth (53).

Two patients (P1 and P3) developed thyroid autoimmunity before tumor, although with different temporal latencies. Both had predisposing conditions for autoimmunity as explained above. In fact, both had reduced numbers of circulating naive T cells, RTEs, and switched B memory. However, these alterations were also present in P2 who never presented thyroid autoimmunity.

Although, in the case of P3, the temporal latency from the onset of autoimmunity suggests that autoimmunity may have contributed to cancer development, we cannot assume the same for P1. In fact, in this case, it is not possible to determine with certainty, given the short temporal latency, whether it was the autoimmunity that induced the tumor or the tumor at an early stage that fostered the autoimmunity.

**Figure 3:**
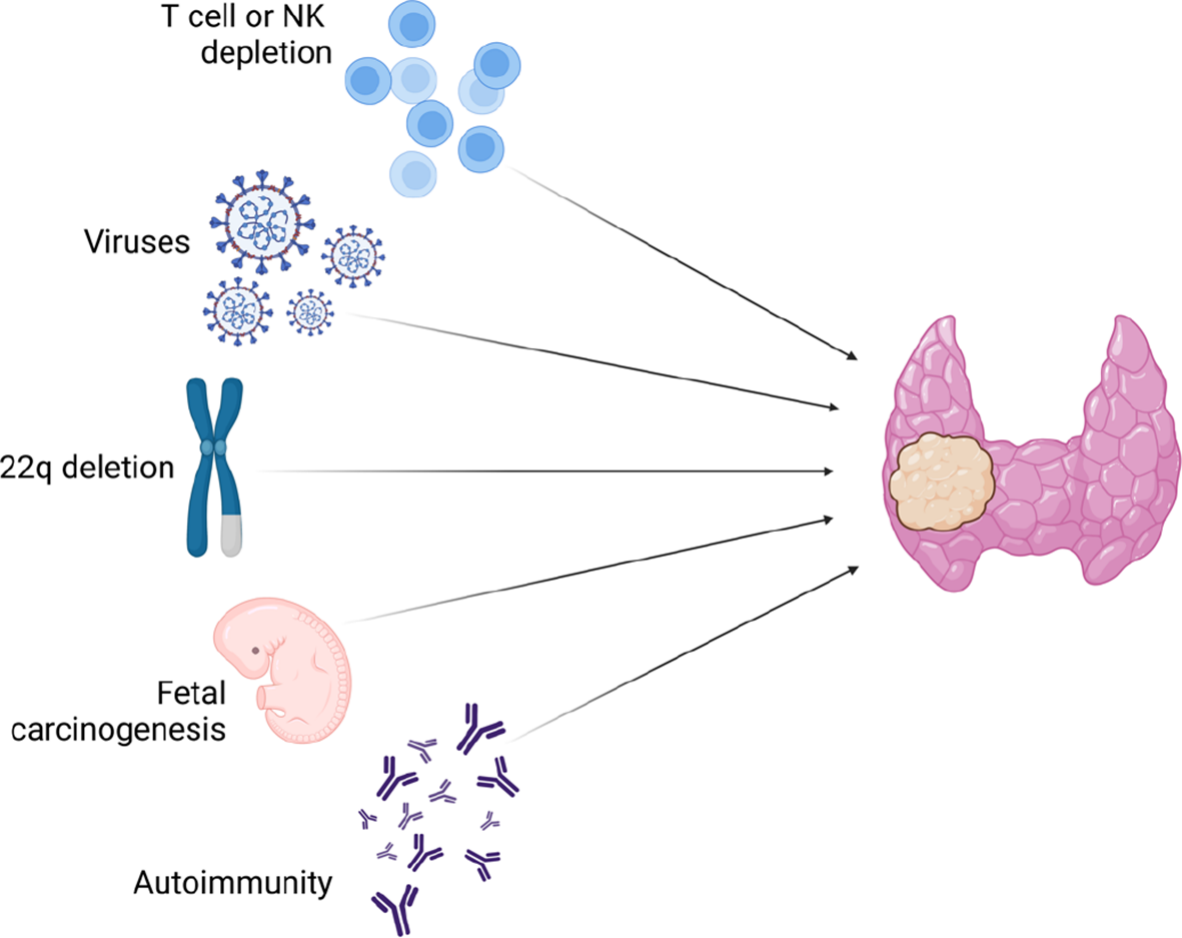
Etiologic hypotheses for increased risk of thyroid neoplasms in 22q11.2DS and DiGeorge-like syndrome patients.

Beyond the various hypotheses presented and depicted in the summary in **Figure 3**, with this work, we draw attention to the fact that prolonged follow-up protocols that include annual or biannual thyroid ultrasound scans not only allow recognition of autoimmune thyroid pathologies before autoantibodies or clinical symptoms become positive but also allow an earlier identification of thyroid nodules, particularly in adolescents with 22q11DS. In fact, all of our cases, both confirmed malignant and suspected, occurred in the age range of 9–15 years. We therefore suggest increasing clinicians’ attention and thyroid ultrasound follow-up in patients within this age group. Early ultrasonographic detection of a thyroid nodule is indeed important because it provides early identification of suspicious features of malignancy, early referral of patients to FNAB, and eventually surgery. Moreover, in case of malignant lesions, it offers the possibility of lowering the risk of metastasis, the need for radiometabolic therapy, and unfavorable outcomes. In addition, it also enables a detailed tracking of any morphological changes in suspicious nodules that cannot undergo early surgery because of a difficult location, as demonstrated for P4. Among the described patients, three patients developed malignant thyroid nodules, and only one of them had a recurrence of disease that required new surgery for lymphadenectomy. In any case, none of these patients presented distant metastases. All of them, reflecting the importance of an early diagnosis, had an excellent prognosis and all of them are steadily disease-free.

## Conclusion

To the best of our knowledge, this work represents the first aggregate of data on the development of thyroid neoplasms in patients with this clinical phenotype. Our data suggest a significantly increased prevalence of neoplasms among patients with 22q11.2DS and highlight how the thyroid may be one of the preferred sites for the development of neoplasms in these patients. Our data also demonstrate that the risk of developing thyroid neoplasms could also be shared by patients with DiGeorge-like syndromes. Thus, in patients with this clinical and immunological phenotype, ultrasound scans of thyroid and parathyroid glands should be performed at the time of diagnosis and then periodically, mostly in adolescence, to ensure early detection and more proper and precocious treatment of neoplasms. A long clinical and ultrasound follow-up for these patients, especially in the presence of more pronounced immunologic changes, is recommended since the risk of neoplasms could persist lifelong.

Anyway, our data are limited to obtain significant conclusions regarding the effective increase of the thyroid cancer risk for 22q11.2DS and DGS-like patients, and more extensive studies will be necessary to be conclusive. A large registry of studies enrolling individuals with 22q11.2DS, regardless of reason or age at diagnosis, would be useful to define the exact risk, especially given the variability in reported cancer types, and begin to understand the precise mechanisms behind.

## Data availability statement

The original contributions presented in the study are included in the article/supplementary material. Further inquiries can be directed to the corresponding author.

## Ethics statement

Ethical review and approval was not required for the study on human participants in accordance with the local legislation and institutional requirements. Written informed consent to participate in this study was provided by the participants’ legal guardian/next of kin.

## Author contributions

All authors participated in the drafting of the article unanimously. All authors read and approved the final article.
